# Supplementary material for: COVID-19 Vaccination Rate under Different Political Incentive: A Counterfactual Trend Approach Using Nationwide Data
Source: Vaccines (Basel). 2023 Jun 25;11(7):1149. doi: 10.3390/vaccines11071149 (PMC10385043; doi:10.3390/vaccines11071149)
Supplement: Supplementary file 1 [file vaccines-11-01149-s001.zip › vaccines-2441205-supplementary.pdf]

Innovation diffusion

Innovation diffusion theory was initially developed to model consumer behaviour when releasing a new product. A proportion of persons wants to buy the new product (or opt for the innovation), and are then followed by imitators. The number of buyers increases, reaches a maximum and decreases when most of the interested people already bought the product. Innovation diffusion theory allows to model the evolution of the weekly vaccination rate using the function (29):

$$V(t) = \frac{K(p+q)\left(1+\frac{\mathrm{p}}{\mathrm{q}}\right)\mathrm{e}^{(\mathrm{p}+\mathrm{q})(\mathrm{t}-\mathrm{t}_0)}}{\left(1+\frac{p}{q}\mathrm{e}^{(\mathrm{p}+\mathrm{q})(\mathrm{t}-\mathrm{t}_0)}\right)^2}$$

$V(t)$ stands for the weekly vaccination rate, with p the innovation coefficient, q the imitation coefficient and K the proportion of the population willing to participate. t_0 is the time at which the innovation (i.e. the vaccination) is accessible.

$V(t)$ is maximum for

$$t_{max} = \frac{\ln\left(\frac{q}{p}\right)}{p+q}$$

T this maximum, It reaches a value of

$$V_{max} = \frac{K(p+q)\left(1+\frac{p}{q}\right)}{4}$$

And has a full width half maximum (ie. at the half height of the peak)

$$\gamma = \frac{1}{p+q}\ln\left(\frac{6+\sqrt{32}}{6-\sqrt{32}}\right)$$

Death excess

The death excess at the EPCI level has been calculated based on the list of all death in France. The national institute of statistics (INSEE) [41] provides the list of all death since 1970, with the death and birth date, death and birth place, and gender. From these files, we calculated the number of deaths per week per EPCI. The expected mortality was then calculated as the mean of the all-cause mortality for the given week and EPCI during the 2017-2019 period. The death excess has been operationalized as the cumulative weekly p score from January 2020 up to the 12th of July 2021

$$p_{score} = \frac{deaths - expected\ deaths}{expected\ deaths} \times 100$$

Political leanings

Table S1. *Opinion of the candidate to the 2017 French presidential election on the vaccination pass.*

**Candidate**	**Pro-pass**
Nathalie Arthaud	No
Fabien Roussel	No
Emmanuel Macron	Yes
Jean Lassalle	No
Marine Le Pen	No
Eric Zemmour	No
Jean Luc Mélenchon	No
Anne Hidalgo	Yes
Yannick Jadot	No
Valérie Pécresse	Yes
Philippe Poutou	No
Nicolas Dupont-Aignan	No

Table S2. Normalized coefficients [Confidence interval] of the multivariate regression predicting the cumulated percentage of the population vaccinated before the implementation of the health pass, for each age category.

term	20-39	40-64	65-75	75+
**Territory**				
Oversea (vs metropolitan)	**-0.4 [-0.5, -0.2]**	**-0.2 [-0.3, -0.08]**	-0.006 [-0.08, 0.06]	0.03 [-0.02, 0.08]
Medium density (ref low)	0.6 [-0.5, 1.7]	**-2.0 [-2.6, -1.3]**	**-4.7 [-5.2, -4.3]**	**-5.2 [-5.6, -4.9]**
High density (ref low)	**0.2 [0.1, 0.23]**	**0.2 [0.1, 0.22]**	**0.1 [0.07, 0.2]**	**0.06 [0.02, 0.1]**
Proportion women	**0.4 [0.3, 0.5]**	**0.3 [0.2, 0.4]**	**0.2 [0.17, 0.3]**	**0.2 [0.1, 0.3]**
**COVID-19**				
Death excess	-0.008 [-0.02, 0.008]	-0.009 [-0.02, 0.006]	**-0.02 [-0.03, -0.002]**	**-0.02 [-0.04, -0.008]**
Hospitalization	0.03 [-0.04, 0.1]	-0.02 [-0.1, 0.05]	-0.02 [-0.09, 0.06]	-0.01 [-0.04, 0.02]
**Socio-economic**				
Poverty	**-0.3 [-0.4, -0.28]**	**-0.3 [-0.33, -0.2]**	**-0.2 [-0.21, -0.1]**	**-0.05 [-0.09, -0.01]**
Population without secondary education	-0.02 [-0.05, 0.006]	**-0.03 [-0.06, -0.003]**	-0.006 [-0.03, 0.02]	-0.002 [-0.02, 0.02]
Inequalities	-0.03 [-0.07, 0.02]	**-0.1 [-0.15, -0.05]**	**-0.1 [-0.2, -0.06]**	**-0.08 [-0.1, -0.05]**
**COVID-19 health access**				
Min distance to vaccination center	**-0.06 [-0.09, -0.04]**	**-0.08 [-0.1, -0.05]**	-0.01 [-0.04, 0.01]	-0.009 [-0.03, 0.02]
Pharmacy and physician density	**-0.02 [-0.04, 0.003]**	0.01 [-0.007, 0.03]	0.01 [-0.007, 0.03]	0.007 [-0.009, 0.02]
Doses available	-0.01 [-0.05, 0.03]	**0.06 [0.02, 0.09]**	**0.04 [0.01, 0.07]**	**0.03 [0.003, 0.05]**
**Politics**				
Abstention	**-0.2 [-0.22, -0.1]**	**-0.1 [-0.2, -0.08]**	**-0.1 [-0.2, -0.05]**	**-0.1 [-0.2, -0.102]**
Result of pro-pass candidate	**0.4 [0.3, 0.396]**	**0.4 [0.3, 0.41]**	**0.2 [0.21, 0.3]**	**0.2 [0.22, 0.3]**

Table S3. Normalized coefficients [Confidence interval] of the multivariate regression predicting the standardized effect of the health pass (the percentage of the population convinced by the health pass that would not have vaccinated otherwise), for each age category.

term	20-39	40-64	65-75	75+
**Territory**				
Oversea (vs metropolitan)	-0.3 [-1.0, 0.4]	**-2.4 [-3.2, -1.5]**	**-1.4 [-1.9, -0.8]**	**-2.1 [-2.6, -1.5]**
Medium density (ref low)	0.05 [-0.02, 0.1]	0.04 [-0.06, 0.1]	**0.3 [0.2, 0.4]**	**0.6 [0.4, 0.7]**
High density (ref low)	0.06 [-0.06, 0.2]	0.07 [-0.09, 0.2]	**0.3 [0.1, 0.5]**	**0.5 [0.3, 0.7]**
Proportion women	0.02 [-0.008, 0.05]	**0.1 [0.06, 0.14]**	**0.2 [0.1, 0.21]**	**0.2 [0.1, 0.25]**
**COVID-19**				
Death excess	**-0.02 [-0.04, 0.004]**	0.002 [-0.03, 0.03]	**0.07 [0.02, 0.1]**	-0.02 [-0.06, 0.03]
Hospitalization	**-0.06 [-0.1, -0.00004]**	0.02 [-0.06, 0.1]	**-0.09 [-0.1, -0.03]**	**-0.09 [-0.1, -0.04]**
**Socio-economic**				
Poverty	**-0.1 [-0.2, -0.07]**	**-0.1 [-0.2, -0.05]**	0.04 [-0.06, 0.1]	0.04 [-0.04, 0.1]
Pop without secondary education	-0.02 [-0.06, 0.01]	-0.03 [-0.08, 0.02]	**-0.09 [-0.1, -0.04]**	**-0.1 [-0.2, -0.08]**
Inequalities	**-0.2 [-0.22, -0.1]**	**-0.1 [-0.2, -0.04]**	**-0.1 [-0.2, -0.07]**	-0.04 [-0.1, 0.03]
**COVID-19 health access**				
Min distance to vaccination center	-0.01 [-0.05, 0.03]	-0.02 [-0.07, 0.03]	**-0.06 [-0.1, -0.002]**	-0.04 [-0.09, 0.01]
Pharmacy and physician density	-0.02 [-0.05, 0.006]	**-0.1 [-0.2, -0.08]**	-0.04 [-0.09, 0.01]	**-0.05 [-0.1, 0.001]**
Doses available	**0.1 [0.05, 0.2]**	0.005 [-0.07, 0.08]	**0.08 [0.02, 0.1]**	0.007 [-0.05, 0.07]
**politics**				
Abstention	**-0.1 [-0.2, -0.02]**	**0.1 [0.02, 0.2]**	**0.2 [0.07, 0.3]**	**0.1 [0.05, 0.2]**
Result of pro-pass candidate	**0.2 [0.18, 0.3]**	**0.06 [-0.005, 0.1]**	**0.1 [0.04, 0.2]**	0.05 [-0.02, 0.1]
